# Zipf’s Law: Balancing Signal Usage Cost and Communication Efficiency

**DOI:** 10.1371/journal.pone.0139475

**Published:** 2015-10-01

**Authors:** Christoph Salge, Nihat Ay, Daniel Polani, Mikhail Prokopenko

**Affiliations:** 1 Department of Computer Science, University of Hertfordshire, Hatfield, United Kingdom; 2 Max Planck Institute for Mathematics in the Sciences, Leipzig, Germany; 3 Santa Fe Institute, Santa Fe, United States of America; 4 Department of Mathematics and Computer Science, Leipzig University, Leipzig, Germany; 5 Complex Systems Research Group, Faculty of Engineering and IT, The University of Sydney, Sydney, Australia; 6 Department of Computing, Macquarie University, Sydney, Australia; University of Illinois-Chicago, UNITED STATES

## Abstract

We propose a model that explains the reliable emergence of power laws (e.g., Zipf’s law) during the development of different human languages. The model incorporates the principle of least effort in communications, minimizing a combination of the information-theoretic communication inefficiency and direct signal cost. We prove a general relationship, for all optimal languages, between the signal cost distribution and the resulting distribution of signals. Zipf’s law then emerges for logarithmic signal cost distributions, which is the cost distribution expected for words constructed from letters or phonemes.

## Introduction

Zipf’s law [1] for natural languages states that the frequency *p*(*s*) of a given word *s* in a large enough corpus of a (natural) language is inversely proportional to the word’s frequency rank. Zipf’s law postulates a power-law distribution for languages with a specific power law exponent *β*, so if *s*_*t*_ is the *t*-th most common word, then its frequency is proportional to

$$\begin{array}{c} p\left(s_{t}\right)∼\frac{1}{t^{\beta }}, \end{array}$$ (1)

with *β* ≈ 1. Empirical data suggests that the power law holds across a variety of natural languages [2], but the exponent *β* can vary, depending on the language and the context, with a usual value of *β* ≈ 2 [3]. While the adherence to this “law” in different languages suggests a underlying common principle or mechanism, a generally accepted explanation for this phenomenon is still lacking [4].

Several papers [5–7] suggest that random texts already display a power law distribution sufficient to explain Zipf’s law, but a detailed analysis [8] with different statistical tests rejects this hypothesis and argues, that there is a “meaningful” mechanism at play, which causes this distribution across different natural languages.

If we reject the idea that Zipfian distribution are produced as a result of a process that randomly produces words, then the next logical step is to ask what models can produce such distributions and agrees with our basic assumptions about language? Mandelbrot [9] models language as a process of producing symbols, where each different symbol (word) has a specific cost. He argues that this cost grows logarithmically for more expensive symbols. He then considers the information of this process, and proves that a Zipfian distribution of the symbols produces the maximal information per cost ratio. Similar, more recent models [10] prove that power laws result from minimizing a logarithmic cost functions while maximising a process’s entropy (or self-information) [11]. But all these cost functions look at languages as a single random process, only optimizing the output distribution and ignoring any relationship between used words and intended meaning. This makes it a questionable model for human language (similar to the models with random text) as it does not account for communication efficiency, i.e., the model is not sensitive to how much information the words contain about the referenced concepts, nor does it offer any explanation on how certain words come to be assigned to certain meanings.

An alternative model by Cancho and Solé [12] follows the original idea of Zipf [1], by modelling the evolution of language based on the principle of least effort, where the assignment of words to concepts is optimized to minimize a weighted sum of speaker and listener effort. While simulations of the model produce distributions which qualitatively resemble power laws, a detailed mathematical investigation [4] reveals that the optimal solution of this model is, in fact, not following a power law; thus, the power law characteristics of the simulation results seems to be an artefact of the particular optimization model utilized.

Thus, to our knowledge, the question of how to achieve power laws in human language from the least effort principle is still not satisfactorily solved. Nevertheless, the idea from [1, 12] to explain power laws as the result of an evolutionary optimization process that minimizes some form of language usage cost remains attractive. In this vein, we present an alternative model for the least effort principle in language: we minimize a cost function consisting of communication inefficiency and an inherent cost for each signal (word). To avoid past pitfalls of statistical analysis when looking for power laws [13], we offer mathematical proof that any optimal solution for our cost function necessarily realizes a power law distribution, as long as the underlying cost function for the signals increases logarithmically (if the signals are ordered according to cost rank). The result generalizes beyond this as we can state a general relationship between the cost structure of the individual signals and the resulting optimal distribution of the language signals.

We should also point out that a power-law often is not the best fit to real data [14]. However, the motivation of our study differs from that of [14] which attempted to find a mechanism, i.e., Random Group Formation (RGF), that fits and, crucially, predicts the data very well—instead, we attempt to find a model formalizing the least effort principle as a mechanism generating power laws.

Another important consideration is that there in general may be multiple mechanisms generating power laws, and one cannot *post hoc* reconstruct necessarily which mechanism resulted in the observed power law. We believe, however, that it is nevertheless useful to develop a mathematically rigorous version of such a mechanism (i.e., the least effort principle) applicable to languages in particular, as it would provide additional explanatory capacity in analyzing structures and patterns observed in languages [15, 16].

The resulting insights may be of interest beyond the confines of power-law structures and offer an opportunity to study optimality conditions in other types of self-organizing coding systems, for instance in the case of the genetic code [17]. The suggested formalization covers a general class of optimal solutions balancing cost and efficiency, with power laws appearing as a special case. Furthermore, the proposed derivation highlights a connection between scaling in languages and thermodynamics, as the scaling exponent of the resulting power law is given by the corresponding inverse temperature (which in general relates the information-theoretic or statistical-mechanical interpretation of a system through its entropy and the system’s thermodynamics associated with its energy).

## 1 Model

We will use a model, similar to that used by Ferrer i Cancho and Solé [12], which considers languages as an assignment of symbols to objects, and then optimizes this assignment function in regard to some form of combined speaker and listener effort. The language emerging from our model is also based on the optimality principle of least effort in communication, but uses a different cost function.

The model has a set of *n* signals *S* and a set of *m* objects *R*. Signals are used to reference objects, and a language is defined by how the speaker assigns signals to objects, i.e. by the relation between signals and objects. The relation between *S* and *R* in this model can be expressed by a binary matrix *A*, where an element *a*_*i*,*j*_ = 1 if and only if signal *s*_*i*_ refers to object *r*_*j*_.

This model allows one to represent both *polysemy* (that is, the capacity for a signal to have multiple meanings by referring to multiple objects), and *synonymy*, where multiple signals refer to the same object. The relevant probabilities are then defined as follows:

$$\begin{array}{c} p\left(s_{i}|r_{j}\right)=\frac{a_{i,j}}{\omega_{j}} \end{array}$$ (2)

where *ω*_*j*_ is the number of synonyms for object *r*_*j*_, that is *ω*_*j*_ = ∑_*i*_ *a*_*i*,*j*_. Thus, the probability of using a synonym is equally distributed over all synonyms referring to a particular object. Importantly, it is also assumed that $p(r_{j})=\frac{1}{m}$ is uniformly distributed over the objects, leading to a joint distribution:

$$\begin{array}{c} p\left(s_{i},r_{j}\right)=p\left(r_{j}\right)\;p\left(s_{i}|r_{j}\right)=\frac{a_{i,j}}{m\omega_{j}}\;. \end{array}$$ (3)

In the previous model [12] each language has a cost based on a weighted combination of speaker and listener effort. The effort for the listener should be low if the received signal *s*_*i*_ leaves little ambiguity as to what object *r*_*j*_ is referenced, so there is little chance that the listener misunderstands what the speaker wanted to say. In the model of Ferrer i Cancho and Solé [12], the cost for listening to a specific signal *s*_*i*_ is expressed by the conditional entropy:

$$\begin{array}{c} H_{R|s_{i}}\left(p\right)\equiv -\sum_{j=1}^{m}p\left(r_{j}|s_{i}\right)\log_{m}p\left(r_{j}|s_{i}\right)\;. \end{array}$$ (4)

The overall effort for the listener is then dependent on the probability of each signal and the effort to decode it, that is

$$\begin{array}{c} H_{R|S}\left(p\right)\equiv \sum_{i=1}^{n}p\left(s_{i}\right)H_{R|s_{i}}\;. \end{array}$$ (5)

Ferrer i Cancho and Solé argue that the listener effort is minimal when this entropy is minimal, in which case there is a deterministic mapping between signals and objects.

The effort for the speaker is expressed by the entropy *H*_*S*_, which is, as the term in Eq (5), bound between 0 and 1, via the log with respect to *n*:

$$\begin{array}{c} H_{S}\left(p\right)\equiv -\sum_{i=1}^{n}p\left(s_{i}\right)\log_{n}p\left(s_{i}\right)\;. \end{array}$$ (6)

Ferrer i Cancho and Solé then combine the listener’s and speaker’s efforts within the cost function Ω_*λ*_ as follows:

$$\begin{array}{c} \Omega_{\lambda }=\lambda H_{R|S}+\left(1-\lambda \right)H_{S}\;, \end{array}$$ (7)

with 0 ≤ *λ* ≤ 1.

It can be shown that the cost function Ω_*λ*_ given by Eq (7) is a specific case of a more general *energy* function that a communication system must minimize [4, 18]

$$\begin{array}{c} \Omega_{\lambda }^{0}=-\lambda I\left(S;R\right)+\left(1-\lambda \right)H_{S}\;, \end{array}$$ (8)

where the mutual information *I*(*S*;*R*) = *H*_*R*_ − *H*_*R*∣*S*_ captures the communication efficiency, i.e. how much information the signals contain about the objects. This energy function better accounts for subtle communication efforts [19], since *H*_*S*_ is arguably both a source of effort for the speaker and the listener because the word frequency affects not only word production but also recognition of spoken and written words [16]. The component *I*(*S*;*R*) also implicitly accounts for both *H*_*S*∣*R*_ (a measure of the speaker’s effort of coding objects) and *H*_*R*∣*S*_ (i.e., a measure of the listener’s effort of decoding signals). It is easy to see that

$$\begin{array}{c} \Omega_{\lambda }^{0}=-\lambda H_{R}+\lambda H_{R|S}+\left(1-\lambda \right)H_{S}=-\lambda H_{R}+\Omega_{\lambda }\;, \end{array}$$ (9)

and so when the entropy *H*_*R*_ is constant, e.g. under the uniformity condition $p(r_{j})=\frac{1}{m}$, the more generic energy function $\Omega_{\lambda }^{0}$ reduces to the specific Ω_*λ*_.

We propose instead another cost function that not only produces optimal languages exhibiting power laws, but also retains the clear intuition of generic energy functions which typically reflect the global quality of a solution. Firstly, we represent the communication inefficiency by the information distance, the Rokhlin metric, *H*_*S*∣*R*_ + *H*_*R*∣*S*_ [20, 21]. This distance is often more sensitive than − *I*(*S*;*R*) in measuring the “disagreements” between variables, especially in the case when one information source is contained within another [22].

Secondly, we define the signal usage effort by introducing an explicit cost function *c*(*s*_*i*_), which assigns each signal a specific cost. The signal usage cost for a language is then the weighted average of this signal specific cost:

$$\begin{array}{c} \sum_{i=1}^{n}p\left(s_{i}\right)c\left(s_{i}\right)\;. \end{array}$$ (10)

This is motivated by the basic idea that words have an intrinsic cost associated with using (speaking, writing, hearing, reading) them. To illustrate, a version of English where each use of the word “I” is replaced with “Antidisestablishmentarianism” and vice versa should not have the same signal usage cost as normal English. The optimal solution considering the signal usage cost alone would be to reference every object with the cheapest signal.

The overall cost function for a language $\Omega_{\lambda }^{c}$ is the energy function trading off the communicative inefficiency with the signal usage cost, with 0 < *λ* ≤ 1 trading off the efforts as follows:

$$\begin{array}{c} \Omega_{\lambda }^{c}\left(p\right)=\lambda \left(H_{S|R}\left(p\right)+H_{R|S}\left(p\right)\right)+\left(1-\lambda \right)\sum_{i=1}^{n}p\left(s_{i}\right)c\left(s_{i}\right)\;, \end{array}$$ (11)

where *p* = *p*(*s*_*i*_, *r*_*j*_) is the joint probability. A language can be optimized for different values of *λ*, weighting the respective costs. The extreme case (*λ* = 0) with only the signal usage cost defining the energy function is excluded, while the opposite extreme (*λ* = 1) focusing on the communication inefficiency is considered. Following the principle of least effort, we aim to determine the properties of those languages that have minimal cost according to $\Omega_{\lambda }^{c}$.

## 2 Results

First of all, we establish that all local minimizers, and hence all global minimizers, of the cost function (11) are solutions without synonyms. Formally, we obtain the following result.

**Theorem 1.** *Each local minimizer of the function*

$$\begin{array}{c} 𝓒\;\to \;\mathbb{R},\;p\;\mapsto \;\Omega_{\lambda }^{c}\left(p\right), \end{array}$$

*where*

$$\begin{array}{c} 𝓒\;:=\;\{p\in 𝓟(S\times R)\;:\;p\left(r_{j}\right)\;=\;\sum_{i}p\left(s_{i},r_{j}\right)=\frac{1}{m}\;\text{for}\;\text{all}\;j\}\;, \end{array}$$

*and* $\Omega_{\lambda }^{c}(p)$ *is specified by the* Eq (11), 0 < *λ* ≤ 1, *can be represented as a function* *f* : *R* → *S* *such that*

$$\begin{array}{c} p\left(s_{i},r_{j}\right)=\{\begin{array}{cc} 1/m & \;\;if\;\;\;s_{i}=f\left(r_{j}\right);\\ 0 & \;\;otherwise. \end{array} \end{array}$$ (12)

The proof is given in Appendix 1. Note that each solution, i.e. each distribution *p* in expression (3), corresponds to a matrix *A* (henceforth called *minimizer matrix*) which is given in terms of function *f* as follows:

$$\begin{array}{c} a_{i,j}=\{\begin{array}{cc} 1 & \;\;\text{if}\;\;\;s_{i}=f\left(r_{j}\right);\\ 0 & \;\;\text{otherwise.} \end{array} \end{array}$$ (13)

The main outcome of this observation is that the analytical minimization of the suggested cost function results in solutions without synonyms—since any function *f* precludes multiple signals *s* referring to the same object *r*. That is, each column in the minimizer matrix has precisely one non-zero element. Polysemy is allowed within the solutions.

We need the following lemma as an intermediate step towards deriving the analytical relationship between the specific word cost *c*(*s*) and the resulting distribution *p*(*s*).

**Lemma 2.** *For each solution* *p* *minimizing the function* $\Omega_{\lambda }^{c}$,

$$\begin{array}{c} H_{R|S}+\frac{1}{\log_{n}m}H_{S}=1\;. \end{array}$$ (14)

The proof follows from the joint entropy representations

$$\begin{array}{c} H_{S,R}=\frac{H_{R|S}}{1+\log_{m}n}+\frac{H_{S}}{1+\log_{n}m} \end{array}$$ (15)

$$\begin{array}{c} \;=\frac{H_{S|R}}{1+\log_{n}m}+\frac{H_{R}}{1+\log_{m}n}\;, \end{array}$$ (16)

noting that for each minimal solution *H*_*S*∣*R*_ = 0, while *H*_*R*_ = 1 under the uniformity constraint $p(r_{j})=\frac{1}{m}$.

**Corollary 3.** *If n = m, H_R∣S_ + H_S_* = 1.

Using this lemma, and noting that each such solution represented as a function *f* : *R* → *S* has the property *H*_*S*∣*R*_ = 0, we reduce the Eq (11) to

$$\begin{array}{c} \Omega_{\lambda }^{c}\left(p\right)=\lambda (1-\frac{1}{\log_{n}m}H_{S}\left(p\right))+\left(1-\lambda \right)\sum p\left(s_{i}\right)c\left(s_{i}\right) \end{array}$$ (17)

$$\begin{array}{c} \;=\lambda +\frac{\lambda }{\log_{n}m}\sum p\left(s_{i}\right)\log_{n}p\left(s_{i}\right)+\left(1-\lambda \right)\sum p\left(s_{i}\right)c\left(s_{i}\right). \end{array}$$ (18)

Varying with respect to *p*(*s*_*i*_), under the constraint ∑*p*(*s*_*i*_) = 1, yields the extremality condition

$$\begin{array}{c} \frac{\lambda }{\log_{n}m}(\log_{n}p\left(s_{i}\right)+1)+\left(1-\lambda \right)c\left(s_{i}\right)-\kappa^{\prime }=0\; \end{array}$$ (19)

for some Lagrange multiplier *κ*′. The minimum is achieved when

$$\begin{array}{c} p\left(s_{i}\right)=\kappa e^{-\beta c(s_{i})}\;, \end{array}$$ (20)

where

$$\begin{array}{c} \beta =\frac{1-\lambda }{\lambda }\log_{n}m\;, \end{array}$$ (21)

$$\begin{array}{c} \kappa =\frac{1}{\sum e^{-\beta c(s_{j})}}\;. \end{array}$$ (22)

In addition, we require

$$\begin{array}{c} c\left(s_{i}\right)=\ln m_{i} \end{array}$$ (23)

for some integer *m*_*i*_ such that ∑*m*_*i*_ = *m*. The last condition ensures that the minimal solutions *p*(*s*_*i*_) correspond to functions *p*(*s*_*i*_, *r*_*j*_) (i.e., minimizer matrices without synonyms). In other words, the marginal probability (20) without the condition (23) may not concur with the probability *p*(*s*_*i*_, *r*_*j*_) that represents a minimizer matrix under the uniformity constraint $p(r_{j})=\frac{1}{m}$.

Under the condition (23), we have $p(s_{i})=\kappa m_{i}^{-\beta }$, while $\kappa =1/\sum m_{i}^{-\beta }$. In general, one may relax the condition (23), specifying instead an upper-bounded error of approximating the minimal solution by any *p*(*s*_*i*_) = *κe* ^−*βc*(*s*_*i*_)^ which would then allow for arbitrary cost functions *c*(*s*).

Interestingly, the optimal marginal probability distribution (20) is the Gibbs measure with the energy *c*(*s*_*i*_), while the parameter *β* is, thermodynamically, the inverse temperature. It is well-known that the Gibbs measure is the unique measure maximizing the entropy for a given expected energy, and appears in many solutions outside of thermodynamics [23–25].

Let us now consider some special cases. For the case of equal effort, i.e. *λ* = 0.5, and *n* = *m*, the solution simplifies to *β* = 1 and $p(s_{i})=\kappa m_{i}^{-1}$, where $\kappa =1/\sum m_{i}^{-1}$.

Another important special case is given by the cost function *c*(*s*_*i*_) = ln *ρ*_*i*_/*N*, where *ρ*_*i*_ is the rank of symbol *s*_*i*_, and *N* is a normalization constant equal to $\frac{n(n+1)}{2m}$ (so that ∑*ρ*_*i*_/*N* = *m*). In this case, the optimal solution is attained when

$$\begin{array}{c} p\left(s_{i}\right)=\frac{\kappa N^{\beta }}{\rho_{i}^{\beta }} \end{array}$$ (24)

with

$$\begin{array}{c} \kappa =\frac{1}{N^{\beta }\sum \rho_{j}^{-\beta }}\;. \end{array}$$ (25)

This means that a power law with the exponent *β*, specified by Eq (21), is the optimal solution in regard to our cost function (11) if the signal usage cost increases logarithmically. In this case, the exponent *β* depends on the system’s size (*n* and *m*) and the efforts’ trade-off *λ*. Importantly, this derivation shows a connection between scaling in languages and thermodynamics: if the signal usage cost increases logarithmically, then the scaling exponent of the resulting power law is given by the corresponding inverse temperature.

Zipf’s law (a power law with exponent *β* = 1) is then nothing but a special case for systems that satisfy ${\text{log}}_{n}\;m=\frac{\lambda }{1-\lambda }$. For instance, for square matrices, Zipf’s law results from the optimal languages which satisfy equal efforts, i.e., *λ* = 0.5. The importance of equal cost was emphasized in earlier works [4, 26]. The exponent defined by Eq (21) changes with the system size (*n* or *m*), and so the resulting power law “adapts” to linguistic dynamics and language evolution in general.

The assumption that the cost function is precisely logarithmic results in an exact power law. If, on the other hand, the cost function deviates from being precisely logarithmic, then the resulting dependency would only approximate a power law—this imprecision may in fact account for different degrees of success in fitting power laws to real data.

In summary, the derived relationship expresses the optimal probability *p*(*s*) in terms of the usage cost *c*(*s*), yielding Zipf’s law when this cost is logarithmically distributed over the symbols.

## 3 Discussion

To explain the emergence of power laws for signal selection, we need to explain why the cost function of the signals would increase logarithmically, if the signals are ordered by their cost rank. This can be motivated, across a number of languages, by assuming that signals are in fact words, which are made up of letters from a finite alphabet; or in regard to spoken language, are made of from a finite set of phonemes. Compare [27], in which Nowak and Krakauer demonstrate how the error limits of communication with a finite list of phonemes can be overcome by combining phonemes into words.

**Fig 1:**
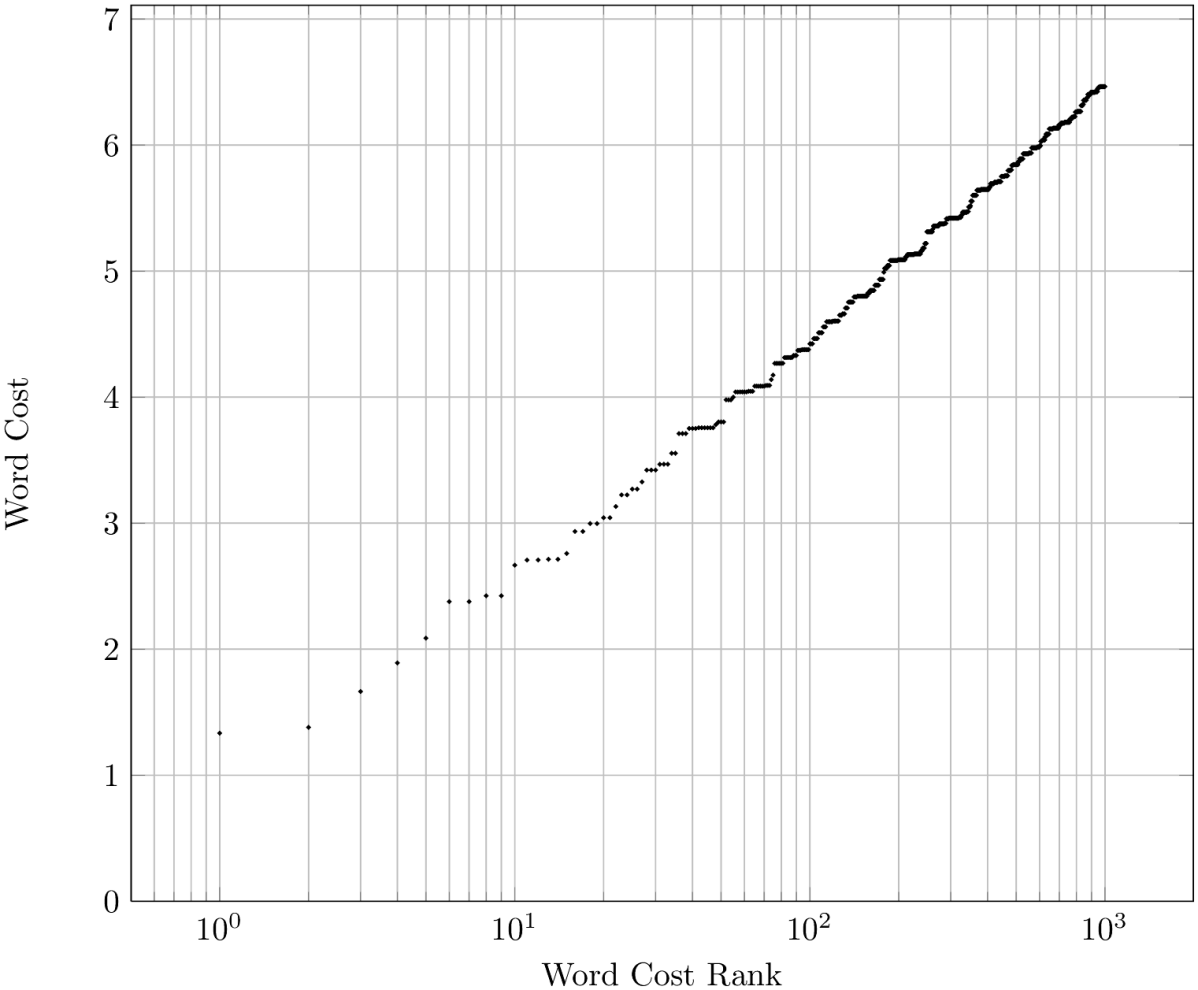
A log-plot of the 1000 cheapest words created from a 10 letter alphabet, ordered by their cost rank. Word cost is a sum of individual letter cost, and letter cost is between 1.0 and 2.0 units.

Lets assume that each letter (or phoneme) has an inherent cost which is approximate to a unit letter cost. Furthermore, assume that the cost of a word roughly equals the sum of its letter costs. A language with an alphabet of size *a* then has *a* unique one letter words which the approximate cost of one, *a* ^2^ two letter words with an approximate cost of two, *a* ^3^ three letter words with a cost of three, etcetera. If we rank these words by their cost, then their cost will increase approximately logarithmically with their cost rank. To illustrate, Fig 1 is a plot of the 1000 cheapest unique words formed with a ten letter alphabet (with no word length restriction), where each letter has a random cost between 1.0 and 2.0. The first few words deviate from the logarithmic cost function, as their cost only depends on the letter cost itself, but the latter words closely follow a logarithmic function. A similar derivation of the logarithmic cost function from first principles can be found in the model of Mandelbrot [9].

This signal usage cost can be interpreted in different ways. In spoken language it might simply be the time needed to utter a word, which makes it a cost both for the listener and the speaker. In written language it might be the effort to write a word, or the bandwidth needed to transmit it, in which case it is a speaker cost. On the other hand, if one is reading a written text, then the length of the words might translate into “listener” cost again. In general, the average signal usage cost corresponds to the effort of using a specific language to communicate for all involved parties. This differs from the original least effort idea, which balances listener and speaker effort [1]. In our model we balance the general effort of using the language with the communication efficiency, which creates a similar tension, as described in [12], between using a language that only uses one signal, and a language that references every object with its own signal. If only communication efficiency was relevant, then each object would have its own signal. Conversely, if only cost mattered, then all objects would be referenced by the same cheapest signal. Balancing these two components with a weighting factor *λ* yields power laws, where *β* varies with changes in the weighting factor. This is in contrast to the model in [12], where power laws were only found in a phase transition along the weighting factor. Also, in [3] Cancho discusses how some variants of language (military, children) have *β* values that deviate from the *β* value of their base language, which could indicate that the effort of language production or communication efficiency is weighted differently in these cases, resulting in different optimal solutions, which are power laws with other values for *β*.

We noted earlier that there are other options to produce power laws, which are insensitive to the relationship between objects and signals. Baek et al. [14] obtain a power law by minimizing the cost function *I*_*cost*_ = −*H*_*S*_ + 〈log *s*〉 + log *N*, where 〈log *s*〉 = ∑*p*(*s*_*i*_)log(*s*_*i*_), and log(*s*_*i*_) is interpreted as the logarithm of the index of *s*_*i*_ (specifically, its rank). Their argument that this cost function follows from a more general cost function *H*_*R*∣*S*_ = −*I*(*S*;*R*) + *H*_*R*_, where *H*_*R*_ is constant, is undermined by their unconventional definition of conditional probability (cf. Appendix A [14]). Specifically, this probability is defined as $p(r|s)=\frac{\delta_{s^{\prime }(r),s}}{sN(s)}$, where *N*(*s*) is the number of objects to which signal *s* refers. This definition not only requires some additional assumptions in order to make *p*(*r*∣*s*) a conditional probability, but also implicitly embeds the “cost” of symbol *s* within the conditional probability *p*(*r*∣*s*), by dividing it by *s*. Thus, we are left with the cost function *I*_*cost*_ *per se*, not rigorously derived from a generic principle, and this cost function ignores joint probabilities and the communication efficiency in particular.

A very similar cost function was offered by Visser [10], who suggested to maximize *H*_*S*_ subject to a constraint 〈log *s*〉 = *χ*, for some constant *χ*. Again, this maximization produces a power law, and again we may note that the cost function and the constraint used in the derivation do not capture communication efficiency or trade-offs between speaker and listener, omitting joint probabilities as well.

Finally, we would like to point out that the cost function −*H*_*S*_ + 〈log *s*〉 is equivalent to the cost function *H*_*R*∣*S*_ − *H*_*S*∣*R*_ + 〈log *s*〉, under constant *H*_*R*_. This expression reveals another important drawback of minimizing −*H*_*S*_ + 〈log *s*〉 directly: while minimizing *H*_*R*∣*S*_ reduces the ambiguity of polysemy, minimizing −*H*_*S*∣*R*_ explicitly “rewards” the ambiguity of synonyms. In other words, languages obtained by minimizing such a cost directly do exhibit a power law, but mostly at the expense of potentially unnecessary synonyms.

There may be a number of reasons for the avoidance of synonyms in real languages. While an analysis of synonymy dynamics in child languages or aphasiacs is outside of scope of this paper, it is worth pointing out that some studies have suggested that the learning of new words by children is driven by synonymy avoidance [28]. As the vocabulary and the word use are growing in children (with meaning overextensions decreasing over time), reducing the effort for the listener becomes more important [29]. Several principles underlying lexicon acquisition by children, identified by Clark [30], emphasize the dynamics of synonymy reduction. For example, the principle of *conventionality* and *contrast* (“speakers take every difference in form to mark a difference in meaning”) combine in providing some precedence to semantic overlaps, leading children to eventually accept the parents’ (more conventional) word for a semantically overlapping concept. The principle of *transparency* explains how a preference to use a more transparent word helps to reduce ambiguity in the lexicon. It has also been recently shown that the exponent of Zipf’s law (when rank is the random variable) tends to decrease over time in children [31]. The study correlated this evolution of the exponent with the reduction of a simple indicator of syntactic complexity given by the mean length of utterances (MLU), and concluded that this supports the hypothesis that the inter-related exponent of Zipf’s law and linguistic complexity tend to decrease in parallel.

Regarding synonyms it should also be noted, that while they exist, their number is usually comparatively low. If we are looking at a natural language, which might have ca. 100.000 words, we will not find a concept that has 95.000 synonyms. Most concepts have synonyms in the single digits, if they have any. The models that look at just the output distribution could produce languages with such an excessive number of synonyms. In our model the ideal solution has no synonyms, but the existing languages, which are constantly adapting, could be seen as close approximations, where out of 100.000 possible synonyms, most concepts have only very few synonyms, if any. As noted earlier, while precise logarithmic cost functions would produce perfect power-law distributions, natural languages do not fit Zipf’s law exactly but only approximately.

These observations support our conjecture that, as languages mature, the communicative efficiency and the balance between speaker’s and listener’s efforts become a more significant driver, and so the simplistic cost function −*H*_*S*_ + 〈log *s*〉 can no longer be justified.

In contrast, the cost function proposed in this paper *H*_*R*∣*S*_ + *H*_*S*∣*R*_ + 〈log *s*〉 reduces to −*H*_*S*_ + 〈log *s*〉 only *after* minimizing over the joint probabilities *p*(*s*, *r*). Importantly, it captures communication (in)efficiency and average signal usage explicitly, balancing out different aspects of the communication trade-offs and representing the concept of least effort in a principled way. The resulting solutions do not contain synonyms, which disappear at the step of minimizing over *p*(*s*, *r*), and so correspond to “perfect”, maximally efficient and balanced, languages. The fact that even these languages exhibit power (Zipf’s) laws is a manifestation of the continuity of scale-freedom in structuring of languages, along the refinement of cost functions representing the least effort principle: as long as the language develops closely to the optima of the prevailing cost function, power laws will be adaptively maintained.

In conclusion, our paper addresses the long-held conjecture that the principle of least effort provides a plausible mechanism for generating power laws. In deriving such a formalization, we interpret the effort in suitable information-theoretic terms and prove that its global minimum produces Zipf’s law. Our formalization enables a derivation of languages which are optimal with respect to both the communication inefficiency and direct signal cost. The proposed combination of these two factors within a generic cost function is an intuitive and powerful method to capture the trade-offs intrinsic to least-effort communication.

## 4 Appendix

**Theorem 1.** *Each local minimizer of the function*

$$\begin{array}{c} 𝓒\;\to \;\mathbb{R},\;p\;\mapsto \;\Omega_{\lambda }^{c}\left(p\right), \end{array}$$

*where*

$$\begin{array}{c} 𝓒\;:=\;\{p\in 𝓟(S\times R)\;:\;p\left(r_{j}\right)\;=\;\sum_{i}p\left(s_{i},r_{j}\right)=\frac{1}{m}\;\text{for}\;\text{all}\;j\}\;, \end{array}$$

*and* $\Omega_{\lambda }^{c}(p)$ *is specified by the* Eq (11), 0 < *λ* ≤ 1, *can be represented as a function* *f* : *R* → *S* *such that*

$$\begin{array}{c} p\left(s_{i},r_{j}\right)=\{\begin{array}{cc} 1/m & \;\;if\;\;\;s_{i}=f\left(r_{j}\right);\\ 0 & \;\;otherwise. \end{array} \end{array}$$ (26)

In order to prove this theorem, we establish a few preliminary propositions (these results are obtained by Nihat Ay).

### 4.1 Extreme points

The extreme points of C are specified by the following proposition.

**Proposition 2.** *The set C has the extreme points*

$$\begin{array}{c} \text{Ext}\left(𝓒\right)\;=\;\{p\in 𝓟(S\times R)\;:\;p\left(s_{i},r_{j}\right)=\frac{1}{m}\;\delta_{f(r_{j})}\left(s_{i}\right)\;\}\;, \end{array}$$

*where* *f* *is a function* *R* → *S*.

***Proof.*** Consider the convex set

$$\begin{array}{c} 𝓣\;=\;\{A={\left(a_{i|j}\right)}_{i,j}\in {\mathbb{R}}^{m\cdot n}\;:\;a_{i|j}\geq 0\;\text{for}\;\text{all}\;i,j\text{,} \end{array}$$

$$\begin{array}{c} \;\;\text{and}\;\sum_{i}a_{i|j}=1\;\text{for}\;\text{all}\;j\} \end{array}$$

of transition matrices. The extreme points of T are given by functions *f* : *j* ↦ *i*. More precisely, each extreme point has the structure

$$\begin{array}{c} a_{i|j}\;=\;\delta_{f(j)}\left(i\right)\;. \end{array}$$

Now consider the map *φ* : T→C that maps each matrix *A* = (*a*_*i*∣*j*_)_*i*,*j*_ to the probability vector

$$\begin{array}{c} p\left(s_{i},r_{j}\right)\;:=\;\frac{1}{m}\;a_{i|j},\;\text{for}\;\text{all}\;i,j. \end{array}$$

This map is bijective and satisfies *φ*((1 − *t*) *A* + *t* *B*) = (1 − *t*) *φ*(*A*) + *t* *φ*(*B*). Therefore, the extreme points of C can be identified with the extreme points of T.

### 4.2 Concavity

Consider the set *S* = {*s*_1_, …, *s*_*n*_} of signals with *n* elements and the set *R* = {*r*_1_, …, *r*_*m*_} of *m* objects, and denote with P(*S* × *R*) the set of all probability vectors *p*(*s*_*i*_, *r*_*j*_), 1 ≤ *i* ≤ *n*, 1 ≤ *j* ≤ *m*. We define the following functions on P(*S* × *R*):

$$\begin{array}{c} H_{S|R}\left(p\right)\;:=\;-\sum_{j}p\left(r_{j}\right)\sum_{i}p\left(s_{i}|r_{j}\right)\log_{n}\;p\left(s_{i}|r_{j}\right)\;, \end{array}$$

and

$$\begin{array}{c} H_{R|S}\left(p\right)\;:=\;-\sum_{i}p\left(s_{i}\right)\sum_{j}p\left(r_{j}|s_{i}\right)\log_{m}p\left(r_{j}|s_{i}\right)\;. \end{array}$$

**Proposition 3.** *All three functions* *H*_*R*∣*S*_, *H*_*S*∣*R*_, *and*

$$\begin{array}{c} \langle c\rangle \;:\;\;p\mapsto \sum_{i}p\left(s_{i}\right)\;c\left(s_{i}\right) \end{array}$$

*that are involved in the definition of* $\Omega_{\lambda }^{c}$ *are concave in* *p*. *Furthermore, the restriction of* *H*_*S*∣*R*_ *to the set C is strictly concave.*

***Proof.*** The statements follow from well-known convexity properties of the entropy and the relative entropy.

**(1)** *Concavity of *H*_*R*∣*S*_*: We rewrite the function *H*_*R*∣*S*_ as

$$\begin{array}{ccc} H_{R|S}\left(p\right) & = & -\sum_{i}p\left(s_{i}\right)\sum_{j}p\left(r_{j}|s_{i}\right)\log_{m}p\left(r_{j}|s_{i}\right)\\ & = & -\sum_{i,j}p\left(s_{i},r_{j}\right)\log_{m}\frac{p(s_{i},r_{j})}{\sum_{j}p\left(s_{i},r_{j}\right)}\\ & = & -\sum_{i,j}p\left(s_{i},r_{j}\right)\log_{m}\frac{p(s_{i},r_{j})}{m\;\frac{1}{m}\sum_{j}p\left(s_{i},r_{j}\right)}\\ & = & -\sum_{i,j}p\left(s_{i},r_{j}\right)\log_{m}\frac{p(s_{i},r_{j})}{\frac{1}{m}\sum_{j}p\left(s_{i},r_{j}\right)}\;+\;1. \end{array}$$

The concavity of *H*_*R*∣*S*_ now follows from the joint convexity of the relative entropy $(p,q)\mapsto D(p\|q)=\sum_{i,j}p(s_{i},r_{j})\;{\text{log}}_{m}\frac{p(s_{i},r_{j})}{q(s_{i},r_{j})}$.

**(2)** *Concavity of* *H_S∣R_*: The concavity of *H*_*S*∣*R*_ follows by the same arguments as in (1). We now prove the strict concavity of its restriction to C.

$$\begin{array}{ccc} H_{S|R}\left(p\right) & = & -\sum_{j}p\left(r_{j}\right)\sum_{i}p\left(s_{i}|r_{j}\right)\log_{n}\;p\left(s_{i}|r_{j}\right)\\ & = & -\sum_{i,j}p\left(s_{i},r_{j}\right)\log_{n}\;\frac{p(s_{i},r_{j})}{p(r_{j})}\\ & = & -\sum_{i,j}p\left(s_{i},r_{j}\right)\log_{n}\;\frac{p(s_{i},r_{j})}{\frac{1}{m}}\\ & = & -\sum_{i,j}p\left(s_{i},r_{j}\right)\log_{n}\;p(s_{i},r_{j})\;-\;\log_{n}\;m. \end{array}$$

The strict concavity of *H*_*R*∣*S*_ now follows from the strict concavity of the Shannon entropy.

**(2)** *Concavity of 〈*c*〉*: This simply follows from the fact that 〈*c*〉 is an affine function and therefore concave and convex at the same time.

With a number 0 < *λ* ≤ 1, we now consider the function

$$\begin{array}{c} \Omega_{\lambda }^{c}\left(p\right)\;=\;\lambda (H_{R|S}\left(p\right)+H_{S|R}\left(p\right))+\left(1-\lambda \right)\;\sum_{i}p\left(s_{i}\right)\;c\left(s_{i}\right)\;. \end{array}$$

From Proposition 3, it immediately follows that $\Omega_{\lambda }^{c}$ also has corresponding concavity properties.

**Corollary 4.** *For* 0 ≤ *λ* ≤ 1, *the function* $\Omega_{\lambda }^{c}$ *is concave in* *p*, *and, if* *λ* > 0, *its restriction to the convex set* C *is strictly concave.*

### 4.3 Minimizers

We have the following direct implication of Corollary 4.

**Corollary 5.** *Let* 0 < *λ* ≤ 1 *and let* *p* *be a local minimizer of the map*

$$\begin{array}{c} 𝓒\;\to \;\mathbb{R},\;p\;\mapsto \;\Omega_{\lambda }^{c}\left(p\right). \end{array}$$

*Then* *p* *is an extreme point of* C.

***Proof.*** This directly follows from the strict concavity of this function.

Together with Proposition 2, this implies Theorem 1, our main result on minimizers of the restriction of $\Omega_{\lambda }^{c}$ to the convex set C.

We finish this analysis by addressing the problem of minimizing $\Omega_{\lambda }^{c}$ on a discrete set. In order to do so, consider the set of 0/1-matrices that have at least one “1”-entry in each column:

$$\begin{array}{c} 𝓢\;:=\;\{\left(a_{i,j}\right)\in {\left\{0,1\right\}}^{n\cdot m}\;:\;\sum_{i}a_{i,j}\geq 1\;\text{for}\;\text{all}\;j\}. \end{array}$$

This set can naturally be embedded into the set T, which we have considered in the proof of Proposition 2:

$$\begin{array}{c} ı:\;\;𝓢\;↪\;𝓣,\;{\left(a_{i,j}\right)}_{i,j}\;\mapsto \;a_{i|j}\;:=\;\frac{a_{i,j}}{\sum_{i}\;a_{i,j}}\;. \end{array}$$

Together with the map *φ* : T→C we have the injective composition *φ* ∘ *ı*. From Proposition 2 it follows that the extreme points of C are in the image of *φ* ∘ *ı*. Furthermore, Corollary 5 implies that all local, and therefore also all global, minimizers of $\Omega_{\lambda }^{c}$ are in the image of *φ* ∘ *ı*. The previous work of Ferrer i Cancho and Sole [12] refers to the minimization of a function on the discrete set 𝓢:

$$\begin{array}{c} {\tilde{\Omega }}_{\lambda }^{c}\;:=\;\Omega_{\lambda }^{c}\circ \varphi \circ ı:\;\;𝓢\;\to \;\mathbb{R}. \end{array}$$

It is not obvious how to relate local minimizers of this function, with an appropriate notion of locality in 𝓢, to local minimizers of $\Omega_{\lambda }^{c}$. However, we have the following obvious relation between global minimizers.

**Corollary 6.** *A point* *p* ∈ C *is a global minimizer of* $\Omega_{\lambda }^{c}$ *if and only if it is in the image of* *φ* ∘ *ı* *and* (*φ* ∘ *ı*)^−1^(*p*) *globally minimizes* ${\tilde{\Omega }}_{\lambda }^{c}$.
